# Impact of surgeon experience on patient outcomes after pelvic and acetabular fracture surgery

**DOI:** 10.1186/s13037-025-00455-x

**Published:** 2025-11-19

**Authors:** Mark Ayoub, Yeng Vue, Emilio Robles, Jetha Tallapaneni, Armen Martirosian

**Affiliations:** Department of Orthopaedic Surgery, University of California, San Francisco, Fresno, USA

**Keywords:** Pelvis, Acetabular surgery, Surgeon experience, Acetabulum outcomes

## Abstract

**Background:**

Emerging data suggests present day surgeons coming out of training are treating fewer pelvic and acetabular injuries in practice than their predecessors. Few studies have investigated the acute effects of this in the perioperative setting, despite the critical condition of many of these patients. Accordingly, there is a need now for studies that examine how surgeon experience affects short-term outcomes in this patient population.

**Methods:**

This is a retrospective cohort performed at a level I trauma center at UCSF- Fresno. Patients who underwent operative fixation for pelvic and/or acetabular fractures treated by a single fellowship-trained orthopaedic trauma surgeon year 1 through year 7 in practice were included. Electronic medical records were retrospectively reviewed for data collection. Yearly time points for surgeon experience were defined and compared. The primary outcome was acute surgical complications within six weeks. Secondary outcome measurements were estimated blood loss (EBL), operative time, transfusion requirements, and unplanned re-operation.

**Results:**

Two hundred and six patients met study criteria. There was a significant difference in complications during year 1 versus after year 1: 16.7% and 4.5%, respectively (*p* = 0.026), and between years 1–2 and after year 2, 13.3% and 3.4%, respectively, (*p* = 0.022). There was no difference in complications after 2 years. Average operative time in the first year was 271.5 minutes, whereas average operative time for years 2-7 was 185.1 minutes (p=0.016). This difference remained significant until after year 4. There was a significant difference in estimated blood loss (EBL) between post-training years 0-2 and years 2+: 505.5 mL and 263.6 mL, respectively (p = 0.039). There was a significance difference in EBL between years 0-3 versus years 3+. There was no statistically significant difference after this. There was no difference in transfusions received or unplanned re-operations at all time points.

**Conclusions:**

There was no difference in complications at most time points. Despite statistically significant decreases in operative times and blood loss over the first five years in practice, pelvis and acetabular surgeries were shown to have significant blood loss and surgical time regardless of experience. Furthermore, there were no differences in transfusions and unplanned re-operations at all time points, suggesting that fellowship-trained surgeons in high-volume centers can safely treat these complex procedures without a clinically significant increase in morbidity early in their careers.

## Background

Pelvic and acetabular surgeries are among the most complex in the field of orthopaedic trauma [1, 2]. Accordingly, surgeon experience can foreseeably result in significant variations in treatment and outcomes. While this has primarily been reported as it relates to long-term outcomes [3–7], there has been minimal investigation of the effect surgeon experience has on these patients in the short-term [8]. This lack of investigation is present despite a significant body of evidence that pelvic-acetabular surgeries are among the most morbid procedures in orthopedics and are performed on some of the sickest patients [2, 9–15]. In addition, a growing body of evidence suggests that the ratio of pelvic and acetabular surgeries per orthopaedic trauma surgeons is decreasing, driven by a combination of more designated trauma centers and an increasing number of fellowship-trained orthopaedic trauma surgeons nationwide [16–19]. Given the complexity of these surgeries, the diminishing case volumes for new providers, and the inherently critical condition of these patients, it is imperative to understand the effect of surgeon experience on acute results.

The purpose of this study was to compare short-term outcomes including immediate surgical complications, estimated blood loss (EBL), transfusions, surgical times, and unplanned re-operations in pelvic-acetabular surgeries based on surgeon experience.

## Methods

### Hypothesis

The authors hypothesized that increasing surgeon experience is associated with lower rates of acute surgical complications, reduced EBL, fewer transfusions, shorter operative times, and fewer unplanned reoperations in pelvic-acetabular surgery.

## Study design

This was an Institutional Review Board approved retrospective cohort study. Patients who underwent operative fixation for pelvic and/or acetabular fractures treated by a single fellowship-trained orthopaedic trauma surgeon in their first seven years of practice at a single level 1 trauma center, Community Regional Medical Center Fresno, CA from September 2017 to January 2025 were included (Current Procedural Terminology codes 27217, 27218, 27226, 27227, and 27228). The primary outcome was incidence of acute surgical complications within six weeks postoperatively, compared across all time points in surgeon experience. Acute surgical complications were defined as infection, wound complications requiring intervention, vascular injury or new neurologic deficits, and loss of reduction or fixation. The secondary outcomes were estimated blood loss (EBL), operative time, transfusion requirements, and unplanned return to the operating room (OR) within six weeks postoperatively.

Time points for surgeon experience were defined as follows: Year 1: 0–12 months in practice, Years 1–2: 0–24 months in practice, Years 1–3: 0–36 months in practice, Years 1–4: 0–48 months in practice, Years 1–5: 0–60 months in practice. Outcomes for each time point were compared to outcomes afterwards, e.g. Year 1 versus year 1+, Years 1–2 versus years 2+, etc.

The inclusion criteria were patients aged 14 years or older who underwent surgical treatment by a single surgeon for a pelvic ring and/or acetabular injury. The exclusion criteria were procedures involving multiple orthopaedic attendings, polytrauma cases where blood loss and case length were not separately recorded for the pelvic-acetabular portion, and patients with multisystemic organ injury requiring surgical intervention by another specialty. Electronic medical records of 235 patients were retrospectively reviewed for data collection.

The patient variables compared between groups included age, sex, substance abuse, comorbidities, polytrauma status, body mass index (BMI), diabetes status, smoking status, fracture morphology and surgical treatment. Fracture morphology was classified as pelvic fractures, acetabular fractures, or combined pelvic ring-acetabulum injuries. Surgical treatment was classified as percutaneous or open.

### Statistical analysis

Data analysis was conducted with SPSS statistical software. Welch’s t-test of unequal variance was used to assess differences in operative times between groups. Student’s t-test was used to compare EBL and transfusions, as well as to compare the operative times for years 1–5 and later.

Chi-square test was used to compare sex distribution, smoking, and substance abuse between groups, Fisher’s exact test was used to compare complications and diabetes, and Student’s t test was used to compare BMI. Welch’s t-test was used to compare age between groups.

Chi square test was used to compare open versus percutaneous surgical treatment, as well as fracture morphology.

## Results

A total of 206 patients met the study criteria. The average age was 41.4 years (range 15–90). There were 71 females (34.5%) and 135 males (65.5%). Patient demographics are outlined in Table 1.

**Table 1:** Demographic data for all patients

Age	41.4 (17–90)
Sex	65.5% Male, 34.5% Female
Acetabular Fractures	43.7% (90)
Pelvic Fractures	47.6% (98)
Combined Pelvic Ring-Acetabulum Injuries	8.7% (18)
Open Surgical Approach	58.3% (120)
Percutaneous Fixation	41.7% (86)
Drug Use	42.6% (87)
Body Mass Index (BMI)	29.7 kg/m^2^
Estimated Blood Loss (EBL)	400.8 cc

Patients in all groups were compared for presence of diabetes, smoking, drug use, average BMI, age, and sex. There was no significant difference between groups in almost all categories, although smoking was significantly greater in year 1 versus after year 1 (30.0% and 12.5%, respectively, *p* = 0.024), and years 1–2 versus after year 2 (25.0% and 11.0%, respectively, *p* = 0.017). Drug use was also found to be higher after year 4 versus before year 4, 49.4% versus 33.3%, respectively, *p* = 0.022, Table 2.

**Table 2:** Demographic data for patients by year

	Age	Sex (%M, %F)	BMI	Diabetes (%)	Smoking (%)	Drug Use (%)
Year 1	37.3(±15.6)	60/40	29.4	16.6	30.0	43.3
After Year 1	42.1(±17.8)	66.5/33.5	30.0	11.9	12.5	39.8
p	0.13	0.15	0.65	0.55	**0.024**	0.84
First 2 Years	40.2(±17.4)	66.7/33.3	29.8	15.0	25.0	48.3
After Year 2	41.8(±17.6)	65.1/34.9	29.9	11.6	11.0	37.0
p	0.54	0.84	0.89	0.65	**0.017**	0.16
First 3 Years	42.8(±18.8)	66.7/33.3	29.3	15.4	19.2	39.7
After Year 3	40.5(±16.7)	64.84/35.16	30.3	10.9	12.5	40.6
p	0.38	0.81	0.34	0.39	0.229	1.00
First 4 Years	42.2(±18.2)	35.0/65.0	29.7	13.7	18.8	33.3
After Year 4	40.3(±16.7)	33.7/66.3	30.1	11.2	10.1	49.4
p	0.54	0.78	0.66	0.68	0.12	**0.022**
First 5 Years	41.2(±17.2)	38.4/61.6	29.8	10.6	16.6	38.4
After Year 5	41.9(±18.7)	23.6/76.4	30.1	18.2	10.9	45.5
p	0.80	0.065	0.81	0.16	0.38	0.42

The proportion of patients operated on was 14.6% in year 1, 14.6% in year 2, 8.7% in year 3, 18.9% in year 4, 16.5% in year 5, 11.2% in year 6, and 15.9% thereafter. A total of 43.7% of patients had acetabular injuries, 47.6% had pelvic injuries, and 8.7% had combined pelvic ring-acetabular injuries. Acetabular and pelvic ring injuries are characterized in Tables 3 and 4, respectively. There were more combined pelvic ring-acetabulum injuries after year 5 compared to years 1–5 (18.2% versus 5.3%, *p* = 0.014).

**Table 3:** Acetabulum and combined pelvic Ring-Acetabulum case characteristics

	*N*	(%)
Posterior Wall	29	27.1
Anterior Column	10	9.3
Transverse	12	11.2
Posterior Column Posterior Wall	4	3.7
Transverse + Posterior Wall	26	24.3
T-shaped	13	12.1
Anterior Column Posterior Hemitransverse	6	5.6
Both Column	7	6.5
Total	107	100

**Table 4:** Pelvis and combined pelvic Ring-Acetabulum case characteristics

	*N*	%
LC Type	56	48.3
APC	49	42.2
Vertical Shear	4	3.4
Combined Mechanism	7	6.0

LC: Lateral Compression; APC: Anterior Posterior Compression; Combined Mechanism: LC/APC + Vertical Shear

There was a significant difference in percutaneous versus open procedures for years 1–4 and after (47.9% percutaneous and 33.7% percutaneous, respectively, *p* = 0.047), and for years 1–5 and after (47.0% percutaneous and 27.3% percutaneous, respectively, *p* = 0.016). There was a significant difference in fracture morphology for years 1–5 and after, 5.3% and 18.2% combined pelvic ring-acetabulum injuries, respectively, *p* = 0.014.

There was a significant difference in the operative time between year 1 and after year 1: 271.5 min and 185.1 min, respectively (*p* = 0.016). This difference remained significant for years 1–2, 1–3, and 1–4 versus after year 2, after year 3, and after year 4, respectively. There was no statistically significant difference after this.

There was a significant difference in EBL during years 1–2 versus after year 2: 505.5 mL and 263.6 mL, respectively (*p* = 0.039). There was a significant difference in EBL between years 1–3 and after year 3: 498.2 mL and 234.0 mL, respectively (*p* = 0.009). There was no statistically significant difference after this.

There was a significant difference in complications during year 1 versus after year 1: 16.7% and 4.5%, respectively (*p* = 0.026), and between years 1–2 and after year 2, 13.3% and 3.4%, respectively, (*p* = 0.022). This difference was no longer statistically significant when comparing years 1–3 and on.

There was no difference in transfusions received at all time points, unplanned reoperations at all time points, or complications after 2 years. The results are outlined in Table 5.

**Table 5:** Pelvic-Acetabular surgery Short-Term outcomes by year in practice

	*n*(%)	Operative Time (minutes)	EBL (mL)	pRBC’s (*n*)	Unplanned Re-operation, *n*(%)	Complications, *n*(%)
Year 1	30 (14.6)	271.5 (±178.4)	493.8 (±720.1)	1.13 (±2.6)	3 (10)	5(16.7%)
After Year 1	176 (85.4)	185.1 (±129.2)	306.8 (±575.1)	0.99 (±2.1)	4 (2.3)	8(4.5%)
p	-	**0.016**	0.115	0.743	0.065	**0.026**
First 2 Years	60 (29.1)	267.3 (±183.2)	505.5 (±844.7)	1.12 (±2.5)	4(6.7)	8(13.3)
After Year 2	146 (70.9)	169.0 (±106.4)	263.6 (±449.1)	0.97 (±2.0)	3 (2.1)	5(3.4)
p	-	**< 0.001**	**0.039**	0.662	0.197	**0.022**
First 3 Years	78 (37.9)	249.4 (±173.0)	498.2 (±830.8)	1.09 (±2.7)	4(5.1)	8(10.3)
After Year 3	128 (62.1)	166.1 (±104.8)	234.0 (±369.0)	0.97 (±1.7)	3(2.3)	5(3.9)
p	-	**< 0.001**	**0.009**	0.695	0.430	0.082
First 4 Years	117 (56.8)	218.6 (±160.2)	376.7 (±705.0)	0.95 (±2.3)	5(4.3)	10(8.5)
After Year 4	89 (43.2)	170.1 (±103.4)	278.0 (±422.3)	1.10 (±1.9)	2(2.2)	3(3.4)
p	-	**0.009**	0.244	0.614	0.701	0.157
First 5 Years	151 (73.3)	200.5 (±149.3)	333.33 (±632.3)	0.89 (±2.1)	6(4.0)	11(7.3)
After Year 5	55 (26.7)	189.8 (±112.7)	336.0 (±506.6)	1.36 (±2.2)	1(1.8)	2(3.6)
p	-	0.630	0.978	0.159	0.678	0.520
All Years	206 (100)	197.7 (±140.3)	334.0 (±598.7)	1.01 (±2.1)	7 (3.4%)	13(6.3%)

EBL, estimated blood loss. pRBC, packed red blood cells

## Discussion

This study showed decreases in complications after year 2, blood loss after year 3, and surgical times after year 4 in practice, for a fellowship-trained orthopaedic trauma surgeon treating pelvic and acetabular fractures. There were no differences in transfusions or re-operations at any time point.

Prior literature has shown a learning curve for surgeons treating acetabulum fractures [20–22] including worse reductions [5, 22] and a higher rate of conversion to arthroplasty with less experience and lower volumes [4]. This is concerning given the evidence of decreasing case volume for orthopaedic trauma surgeons [16]. There are almost no studies examining acute outcomes in the perioperative setting, despite pelvic-acetabular surgeries being some of the more invasive procedures in orthopaedics performed on some of the sickest patients [2, 9–15] with the highest risk of bleeding and mortality [23]. One large series reviewed the short- and medium-term effects of surgeon experience on outcomes and reported that 2.4 years in practice were needed to reach 50% peak performance with respect to re-operation [3]. However, this study included multiple surgeons, where other confounding factors may have been a significant factor, and it did not directly compare surgical time, blood loss, or transfusions. Caudron et al. reported that with 46 anterior acetabular fracture cases over 5 years, both short- and long-term outcomes did not improve. They suggested that the high (37%) complication rate may reflect the steep learning curve associated with acetabular surgery [24].

This study is consistent with other studies that have shown that a learning curve exists for pelvis and acetabulum surgery. Specifically, short-term outcomes seem to follow this trend. Notably, while there was a statistically significant decrease in surgical time and blood loss with more years in practice, this difference may not be clinically significant. Even after year 5, the average surgical time still exceeded 3 h and the average blood loss remained over 300 cc, indicating that these are inherently more complex, invasive, and morbid procedures, regardless of surgeon experience. This is reflected in the lack of difference in transfusions at all time points despite longer surgeries initially.

Complications in this series included nerve palsies, infections, and postoperative dislocation. There were no arterial or major venous injuries encountered. Nerve injuries were primarily sciatic nerve neuropraxia and were noted in patients with difficult to retrieve intraarticular fragments. The surgeon attributed this to simultaneous stretch of the nerve from axial traction used to distract the joint and from the sciatic nerve retractor used for visualization. In subsequent cases where traction was needed for loose body removal, a retractor was placed superficial to the nerve during hip joint distraction. Additionally, for all posterior approaches, assistants were directly instructed *prior* to the case to relax the sciatic nerve retractor forward whenever possible.

There was one instance of an early re-dislocation in a comminuted posterior wall fracture-dislocation attributed to plate positioning. In later cases, small diameter K wires were used to probe and define the lateral margin of the posterior wall, allowing for more peripheral plate placement.

Multiple adjustments were made that improved efficiency. These strategies included using retractor systems anchored to bone with wires, requesting OR staff familiar with orthopedic equipment, positioning morbidly obese patients with isolated posterior wall fractures in the lateral decubitus position, and pre-contouring plates on a pelvic model preoperatively for sterilization and intraoperative use.

While efforts to maximize efficiency were necessary, in some critically ill patients the emphasis on limiting operative time and morbidity occasionally affected reduction quality. The authors found it helpful to adopt the perspective that even the sickest ICU patients could make a full recovery and therefore warranted optimal reductions. Additionally, in polytrauma patients or those requiring multiple surgical approaches, the surgeon found staging the pelvic-acetabular procedure helpful to allow resuscitation between operations.

Finally, the primary surgeon found reviewing these cases with more experienced mentors, as well as co-scrubbing their mentors’ cases, to be an invaluable enhancement to their practice.

It is worth noting that this study was conducted at a high-volume center for pelvis-acetabulum fractures, and results may not be generalizable to lower-volume centers or surgeons. In general, the acetabular case volume per surgeon has declined substantially in recent years, and especially so compared to the days of Joel Matta and Émile Letournel [25, 26]. Given this dilution of acetabular fracture cases, referral to higher volume centers, close collaboration with experienced mentors, and continued attention to the work of pioneers in the field are essential for achieving surgical goals and optimizing both short- and long-term outcomes.

There are several limitations to this study. First, this was a single surgeon retrospective series. While focusing on one surgeon may eliminate some confounding factors, controlling for differences in skill level, prior training, personality, etc., it may also limit its generalizability to all providers since different providers have different inherent skills, training, and experience that can uniquely affect their results. For example, among anterior acetabular approaches some surgeons are more comfortable with the modified Stoppa (intrapelvic) approach, others prefer the ilioinguinal approach, and few are equally proficient with both. This variability can influence both short- and long-term outcome, as surgical versatility and adaptability are important factors in patient care. Second, the sample size was a significant limitation. Given the wide range of pelvis and acetabular injuries, this results in a smaller number of patients in each subgroup that precludes direct comparisons for individual injury patterns. Third, estimation of blood loss is known to be highly provider dependent and generally imprecise [27–29]. Blood loss can also be influenced by multiple factors, including anticoagulation use preoperatively, patient co-morbidities, use of tranexamic acid (TXA), anesthesia, systemic injuries, and coagulopathy. Anticoagulation use and poorly controlled blood pressure can increase blood loss; TXA has been shown to decrease transfusion rates [30] and potentially blood loss in pelvis-acetabular surgeries [31, 32]. These concerns are somewhat mitigated in this study by having a single surgeon for all cases and by the consistent hospital setting. Specifically, this Level I trauma center has a protocol of initiating prophylactic enoxaparin for all admitted patients and the treating surgeon adopted an early protocol of administering IV TXA for all open cases prior to incision and requesting permissive hypotension whenever possible from the anesthesia provider. While these factors do not guarantee accurate EBL quantities, they likely improve consistency across cases, allowing for more meaningful comparisons. Finally, this investigation was limited to immediate short-term sequalae and did not examine patients’ radiographic or long-term clinical outcomes; these were outside the scope of the study.

## Conclusions

For early career pelvic and acetabular surgeries there were no significant differences in complications after two years, and no significant differences in unplanned reoperations and transfusions at all time points. While there were decreases in operative time and blood loss over the first five years in practice, pelvic and acetabular injuries were shown to require large surgeries with significant surgical time and blood loss regardless of surgeon experience.

Critical lessons learned early in practice included multiple measures to improve efficiency, minimization of unnecessary retraction involving the sciatic nerve, more deliberate and peripheral plate placement for posterior wall fractures, staging surgery whenever possible for polytrauma and ICU patients, and regular engagement with mentors.

The authors believe these findings suggest that fellowship-trained surgeons at high volume pelvis-acetabulum centers can safely perform these difficult procedures without a clinically significant increase in morbidity early in their careers. For orthopaedic trauma surgeons practicing at lower-volume centers, caution should be exercised treating these injuries and referral to higher-volume surgeons should be considered given the inherent complexity of these surgeries and risk for complications.

## Data Availability

The datasets used and/or analyzed during the current study are available from the corresponding author on reasonable request.

## Abbreviations

BMI: Body Mass Index
EBL: Estimated Blood Loss
OR: Operating Room
LC: Lateral Compression
APC: Anterior Posterior Compression
pRBC: packed red blood cells
ICU: Intensive Care Unit
TXA: Tranexamic Acid
